# Editorial: Bioactive Compounds from Microbes

**DOI:** 10.3389/fmicb.2017.00392

**Published:** 2017-03-08

**Authors:** Roberto Mazzoli, Katharina Riedel, Enrica Pessione

**Affiliations:** ^1^Laboratory of Microbial and Applied Biochemistry and Proteomics, Department of Life Sciences and Systems Biology, University of TurinTurin, Italy; ^2^Department for Microbial Physiology and Molecular Biology, Institute of Microbiology, University of GreifswaldGreifswald, Germany

**Keywords:** gut-brain axis, antitumor activity, viral immune-escape, Bacteriocins, human-microbes cross talk

Microorganisms are ubiquitous and essentially interact with all the other organisms present in the biosphere, sometimes creating a network of signals that constitutes the basis for life on the Earth. Clarifying the nature of these molecular signals, their targets and the pathways underlying their production, constitutes the essential pre-requisite for deciphering inter-kingdom communications, adaptive responses and systems biology (Nicholson et al., 2012; O'Mahony et al., 2015; Cani and Knauf, 2016). The insights acquired in the last two decades about human microbiota, and its fundamental role in maintaining a healthy physiological status, have opened the way to understanding the complex reciprocal talk between bacteria and humans (Hughes and Sperandio, 2008; Lyte and Freestone, 2010; Mayer, 2011; Cryan and Dinan, 2012; Mazzoli, 2014; Halang et al., 2015; Kelly et al., 2015). In parallel with these aspects, new bioactive molecules from the microbial world that interact with different cellular models continue to be discovered.

The times and the technological advances start now to be suitable for intercellular/interorganism communication to be studied at the molecular level. The aim of the present topic issue is to try to describing the mediator molecules of a network of signals which is still largely underexplored and underexploited.

As an example, some soil bacteria (such as *Serratia* spp.) can have antagonistic actions toward worms and the molecule involved, *zeamine*, is effective against yeasts and other biological systems, as well. *Prodigiosin*, the well-known pigment produced by the marine bacterium *Vibrio ruber*, has a broad antimicrobial spectrum and induces autolytic activity in the target cells (i.e., *Bacillus subtilis*). *Lantibiotics* are class I bacteriocins produced by Gram-positive bacteria that can be bioengineered to both enhance their effectiveness against a larger number of bacterial strains and to improve their stability during the gastric transit that is by rendering them protease-resistant. These overall data open new possibilities for antibiotic therapy in a period in which the phenomenon of antibiotic resistance is considered as a major threat to public health (World Health Organization, 2014) since it is widespread in pathogenic, commensal, and food bacteria (Laxminarayan et al., 2013). Furthermore, the appearance of multiresistant bacterial strains (the so-called superbugs), often causing death, clearly constitutes a serious problem to be solved. Exploiting the microbial world and its huge potential in finding new antimicrobial drugs is an urgent concern and some chapters of this topic issue deal with these aspects.

Other interesting molecules are produced by cytomegalovirus-infected cells: these compounds of viral origin (essentially *proteins*) can promote virus dissemination, persistence, and pathogenesis by counteracting host innate and adaptive immune responses. Conversely, some beneficial microbes, like Lactic Acid Bacteria (LAB) and Bifidobacteria can modulate the immune system controlling inflammation by means of proteins and non-proteinaceous compounds (Pessione, 2012).

Several microbiota-derived compounds can contribute to control host physiological and pathological states: Metabolomic profiling of gut bacteria can allow to decipher several molecules (among which *short chain fatty acids*, for instance those produced by bacteria belonging to the clostridial cluster IV and XIV, vitamins and aromatic compounds) controlling cholesterol synthesis, obesity, cardiovascular diseases, metabolic syndrome. Curiously, some bioactive *peptides* or amino acids can be delivered by food in which LAB are present as fermentation starters. These bacteria can decrypt peptides from food proteins, which have anti-oxidant or metal chelating function, immunomodulatory properties as well as peptides controlling hyperglycemia, hypercholesterolemia, hypertension, cell cycle, apoptosis, and even having a refolding action on damaged proteins. An interesting tannin-degrading activity of bacteria (e.g., *Streptococcus* spp. and *Fusobacterium* spp.) can generate *gallic acid and pyrogallol*, both having an anti-carcinogenic role. Finally, microbial-derived *amines* can modulate a series of patho/physiological functions such as allergy, smooth muscle relaxation, anxiety, appetite, depression (Pessione et al., 2005). LAB are good producers of gamma-amino butyric acid (GABA) (Laroute et al., 2016) that contributes to gut-to-brain signaling through different pathways involving enteric neurons, entero-endocrine cells and immune cells. Yeasts, mainly *Saccharomyces cerevisiae*, can convert tryptophan into melatonin and serotonin that are informational molecules related to circadian rhythm but also promising agents for the prevention of neurodegenerative diseases.

This intense network of molecules, allowing communication among bacteria, viruses, and eukaryotic cells has evolved to guarantee optimal life in different ecological niches to each component of the ecosystem and is based upon effector-receptor model. In-depth knowledge of all these biochemical signals, as well as the underlying finalism are still far to be fully elucidated. Nevertheless, some mechanistic aspects highlighted in the present topic issue can open new perspectives in medicine but can also shed light on evolution strategies.

## Author contributions

EP wrote the manuscript which was then reviewed by RM and KR.

### Conflict of interest statement

The authors declare that the research was conducted in the absence of any commercial or financial relationships that could be construed as a potential conflict of interest.
